# Commentary: Rethinking pathways to well-being: the function of faith practice in distress alleviation among displaced Muslim women affected by war

**DOI:** 10.3389/fpsyt.2025.1717898

**Published:** 2025-12-01

**Authors:** Aamer Aldbyani

**Affiliations:** General Education Department, Shandong Xiehe University, Jinan, China

**Keywords:** islamic mindfulness, faith-sensitive coping, muslim women, psychosocial interventions, mindfulness

## 1 Introduction

This article presents an expansive commentary on Rutledge’s (1) mixed-methods investigation into the role of faith practices in the psychological well-being of displaced Muslim women in northern Iraq. Rutledge’s study makes an important contribution by demonstrating that religious devotion—particularly daily prayer, Qur’an recitation, and fasting—serves as a primary channel through which women interpret adversity and sustain hope, and that perceptions of divine care mediate the relationship between religious commitment and mental-health outcomes. These insights underscore the importance of considering faith as a central dimension of humanitarian psychosocial support.

The present commentary extends this contribution by highlighting an aspect not explicitly addressed in the original research: the contemplative dimension of Islamic devotional life. Practices such as *khushūʿ* in prayer, *dhikr* (remembrance of God), *murāqabah* (self-awareness before God), and *tafakkur* (reflective contemplation) constitute long-standing, culturally embedded disciplines that foster attentional presence, emotional regulation, and ethical–spiritual orientation. Recognizing these practices as integral to religious coping can deepen understanding of the pathways through which faith supports resilience during displacement.

By framing these contemplative elements as complementary to, rather than separate from, core religious observances, this expansive commentary broadens the interpretive scope of Rutledge’s findings and suggests practical implications for designing culturally congruent mental-health and psychosocial interventions for conflict-affected Muslim communities.

This commentary is intended as a complementary extension to Rutledge’s (1) foundational empirical work. It seeks to widen the analytical lens through which the reported faith-sensitive coping mechanisms can be understood by framing them within the long-standing Islamic tradition of contemplative practices. The aim is not to correct or diminish the original findings but to enrich their interpretation and suggest potential avenues for faith-sensitive psychosocial support that resonate with the participants’ existing spiritual frameworks.

## 2 Acknowledging the contribution

Rutledge’s (1) mixed-methods investigation makes a substantive contribution to humanitarian mental-health research by demonstrating that faith-sensitive engagement with religious practices—especially prayer, Qurʾān recitation, and fasting—constitute central pathways of coping for displaced Muslim women affected by the ISIS conflict. The study reports that perceptions of divine care versus neglect mediate links between faith practice and psychological outcomes. By documenting this dynamic within a gender- and faith-specific displacement context, the research advances understanding of the culturally embedded mechanisms through which religious commitment influences adaptation to prolonged adversity.

Recent scholarship corroborates Rutledge’s (1) findings by demonstrating that core devotional acts—especially daily prayer, Qurʾān recitation, and fasting—serve as primary psychological resources for Muslim women experiencing war-related displacement, offering meaning, connection to God, and hope in protracted adversity (2–4). Evidence from multiple contexts also shows that positive perceptions of God’s responsiveness are linked with lower distress, whereas perceptions of divine punishment or neglect predict poorer mental-health outcomes (5, 6). Additional empirical evidence has now emerged from within an active humanitarian context.

A critical caveat must be noted regarding the evidence base. While Rutledge’s (1) study is situated within a large-scale humanitarian response in a low- and middle-income country (LMIC), much of the existing research on Islamic contemplative practices originates from non-humanitarian settings, such as clinical, university, or migrant populations in high-income countries (HICs). It is important to note that the transferability of findings from these contexts to active humanitarian emergencies requires careful consideration. However, emerging evidence from comparable fragile settings, such as recent work with internally displaced persons in Yemen (7), begins to provide empirical support for the relevance of these practices in crisis-affected populations.

However, even within these more recent field studies conducted in humanitarian response settings, the representation of women remains lower than that of men. This limitation warrants caution in generalizing the findings to displaced women who often face more restrictive gender norms and barriers to participation, and it reinforces Rutledge’s call for further research amplifying women’s voices in camp and displacement contexts. The Yemeni study, undertaken among internally displaced persons living within a Level 3 humanitarian emergency, demonstrated that mindfulness facets and distress tolerance significantly predicted trauma recovery. This field-based evidence confirms that contemplative faith practices can function as adaptive coping mechanisms even under extreme humanitarian conditions and offers a contextual foundation for the practical recommendations that follow.

Research among Muslim refugee and immigrant women in Western settings further highlights that prayer and religiously framed mindfulness practices support a sense of agency, belonging, and reductions in anxiety and depression (8, 9). In addition, studies on culturally adapted mental health care, along with qualitative findings from older Iranian Zoroastrians, indicate that incorporating religious and spiritual elements into psychosocial interventions can improve their relevance and effectiveness among diverse populations, including displaced Muslim women (10).

## 3 Making the contemplative dimension explicit in humanitarian faith-sensitive practice

While Rutledge (1) documents the protective role of prayer, Qurʾān recitation, and fasting, the present commentary specifies a structured contemplative dimension within these acts, acknowledging their devotional origin while examining their psychosocial functions. Islamic mindfulness is an indigenous tradition expressed through *khushū*ʿ in ritual prayer, *dhikr* (remembrance of God), *murāqabah* (self-monitoring under divine awareness), and *tafakkur* (reflective meditation), which together cultivate attentional presence, emotional regulation, and self-restraint before God (11–13). Recognising this contemplative dimension clarifies and deepens Rutledge’s humanitarian reading of women’s practices and supports the inclusion of religious practices in humanitarian and MHPSS programmes not only as ritual obligations but also as embodied attentional strategies that strengthen cognitive and emotional regulation in crisis contexts.

Complementary practices such as *tafakkur* (reflective contemplation) and *murāqabah* (self-accountability before God) are integral to the Islamic therapeutic paradigm and offer culturally grounded alternatives to imported models of meditation. In this commentary, the term “Islamic therapeutic paradigm” refers to a contemporary analytical lens rather than a classical category. Practices such as *khushū*ʿ, *dhikr*, *murāqabah*, and *tafakkur* originate as devotional and contemplative acts grounded in worship, not as clinical techniques. These practices are devotional in origin; their therapeutic function emerges only when contextualized within contemporary psychosocial frameworks, without stripping their spiritual meaning. These practices foster a theocentric rather than merely self-referential mode of awareness, shaping moral orientation and adaptive coping in adversity (14).

Historically and theologically, these practices are embedded in Qurʾānic exhortations to remembrance and reflection and in classical discussions of *iḥsān* and *taqwā*. In many Muslim communities, *dhikr* and attentive prayer are routine elements of daily devotion, while reflective *tafakkur* and self-accounting (*murāqabah*) are widely taught in sermons and study circles. Their familiarity among observant Muslims supports their feasibility as culturally congruent components of psychosocial care when presented in ways that respect local religious authority and custom.

This conceptual and cultural foundation frames mindfulness as a historically embedded and religiously meaningful practice, providing the basis for its contemporary therapeutic adaptation and motivating the subsequent consideration of empirical evidence.

Conceptually, the preceding section outlined the Islamic therapeutic framework; empirically, the following section demonstrates supporting evidence.

## 4 Islamic mindfulness as a continuation of devotional practice and positive reappraisal

Although most of the empirical studies reviewed in this section were conducted in non-humanitarian contexts—such as clinical, university, or migrant settings in high-income countries—the emotion-regulation functions they identify help to illuminate Rutledge’s observations from camp-based humanitarian responses, while warranting caution about direct transferability. In line with the conceptual foundations outlined above, empirical evidence provides further support for the integration of culturally embedded mindfulness practices. Evidence from neuroimaging research supports this perspective. In a functional MRI study, Saraei et al. (15) found that Islamic *dhikr*, contemplation of God, and body-scan meditation each activated neural networks associated with emotion regulation and attentional control, including the prefrontal cortex and hippocampal regions, in ways comparable to certain forms of Buddhist meditation. A quasi-experimental study among older adults in residential care found that mindful *dhikr* practice significantly reduced depressive symptoms and enhanced psychological calm (16).

Prayer practices that incorporate mindful *khushūʿ*—characterized by focused attention and intentional breath control—have been shown to engage neural pathways associated with emotion regulation and serotonergic activity. Through slow, rhythmic breathing and moment-to-moment awareness, such practices may modulate the parasympathetic nervous system and enhance mental and physical well-being (17). Further research demonstrates that the quality of attentional focus during prayer strengthens neural networks associated with self-regulation and cognitive control (11).

Across diverse populations, engagement in religious mindfulness practices—particularly *dhikr* and prayer performed with conscious presence—has been associated with improved autonomic balance and lower levels of stress, anxiety, and depressive symptoms (18, 19). These findings suggest that integrating Islamic mindfulness into culturally adapted faith-sensitive frameworks can strengthen emotional regulation and resilience among Muslims affected by conflict and displacement. Analyzing these devotional practices as potential sources of structured attentional discipline also motivates the subsequent consideration of culturally adapted mindfulness programmes that align methodologically with Islamic forms of presence before God.

Empirical studies and programmatic evaluations indicate that Islamic devotional practices performed with mindful attentiveness support attentional regulation, positive reappraisal, and stress reduction, consistent with the conceptual account outlined above. Rutledge’s (1) analysis shows that many displaced Muslim women linked their distress to perceived shortcomings in religious practice and to doubts about divine concern. Islamic mindfulness is not an alternative to devotion but a continuation of it, enabling believers to reappraise hardship through present-moment awareness, trust, and surrender to God’s decree (11, 20).

Empirical evidence supports this conceptual link. Doufesh et al. (21) demonstrated that focused prayer is associated with neurophysiological markers of attentional regulation, and Purwanto et al. (22) reported that *dhikr*-based interventions reduce stress and support adaptive coping. Yousaf et al. (23) developed the Mindfulness during Worship Scale (MWS), empirically demonstrating that attentional focus, presence, and absorption are core components of worship and prayer. The validated three-factor model reflects structured attentional engagement, emotional awareness, and spiritual immersion. Henry (24) conceptualizes Islamic prayer as a structured psychospiritual practice that integrates intentional focus with spiritual meaning, enhancing psychological resilience and attentional regulation. A qualitative study by Callender et al. (8) with Muslim migrant women in the United States described daily prayer as an attentional exercise with protective effects on mental health.

Physiological evidence also supports this perspective. Nayef (25) found that Qurʾān recitation significantly enhanced heart rate variability and promoted slower, more regulated breathing patterns among university students, indicating improved autonomic regulation and self-regulatory capacity during devotional practice. In a randomized controlled trial, Hanafi et al. (26) demonstrated that listening to Qurʾān recitation significantly reduced physiological stress markers, including electromyogram and skin conductance levels, when assessed via biofeedback. These findings suggest that Qurʾānic audio exposure may enhance psychophysiological regulation in stress-inducing contexts. Regarding positive cognitive reappraisal, Khan et al. (27) developed the Islamic Tawakkul Scale, capturing an active striving combined with trust in God’s outcome, which was inversely associated with psychological distress. In a study of Palestinian adults, Mahamid and Bdier (28) found that positive religious coping—characterized by trust, meaning, and hope—was significantly associated with lower levels of stress and depressive symptoms. These findings highlight the protective role of religious coping strategies in psychologically taxing environments.

Further evidence on intervention feasibility comes from Sarfraz et al. (29), who conducted a culturally adapted online mindfulness program for university students in Pakistan. The pilot randomized controlled trial demonstrated reduced stress and improved psychological well-being among participants, with positive feedback on the program’s acceptability and relevance. A scoping review by Kamarulbahri et al. (30) concluded that Islamic-adapted mindfulness programs yield better mental health outcomes than standard, non-adapted approaches. Collectively, these findings suggest that attentional presence and moment-to-moment awareness are integral to Islamic devotional practices, and that prayer, *dhikr*, and related practices facilitate positive cognitive reappraisal and stress reduction. This evidence reinforces the conceptual and practical integration of Islamic mindfulness into mental-health and psychosocial programs for displaced and refugee Muslim women.

## 5 Ethical–spiritual dimensions linking mindful presence to perceived divine care

A core theme in Rutledge’s (1) findings is the mediating role of perceived divine care in shaping mental-health outcomes among displaced Muslim women. In this context, ethical–spiritual disposition denotes a value-laden mode of attentiveness shaped by *iḥsān* (excellence in worship) and *taqwā* (God-conscious restraint) that orients appraisal, emotion, and behavior toward accountability before God. Intentionality refers to *niyyah*—the explicit, purposive orientation that structures the act of worship and the mindful monitoring of one’s inner states (*murāqabah*), thereby linking devotion with self-regulation and coping. Defined in this way, the ethical–spiritual disposition and intentionality clarify how mindful presence in worship can influence coping mechanisms previously discussed in this commentary, including cognitive reappraisal and the perception of divine care.

Contemporary literature indicates that mindful presence in Islamic devotional practices is not merely a cognitive skill but an ethical–spiritual disposition that enhances the perception of divine care and functions as a psychological resource in coping with stress. Ahmad and Suyuthi (31) argue that mindful engagement in prayer, rooted in Islamic concepts like intentionality, muraqabah, and calm presence, cultivates a sustained awareness of God’s nearness. This ethical–spiritual attentiveness mirrors the Qurʾānic principle of *iḥsān*—worshipping as though seeing God—and serves to frame worship as a pathway to spiritual composure and psychological resiliency.

Qualitative research by Callender et al. (8) with Muslim migrant women in the United States reported that prayer and *dhikr*, when individuals report a sense of mindful presence, fostered a felt sense of God’s nearness and care, supporting psychological endurance in displacement settings. Abu-Raiya and Pargament (32) identified that positive religious coping strategies—such as *tawakkul* (trustful reliance on God) and gratitude—were significantly associated with greater psychological well-being among Muslim parents facing the severe stress of having a child with cancer. These coping styles appeared to enhance meaning-making, emotional regulation, and perceived social support.

From a mental-health perspective, Elqaq (33) found that religion-based mindfulness meditation among Palestinian Muslims promoted positive reappraisal of stressful experiences, reduced perceived threat, and enhanced the sense of divine care. These outcomes reflect Pargament’s (34) model of positive religious coping, where viewing God as present and supportive is linked to emotional resilience. This may also explain observations by Rutledge that women who viewed God as distant reported worse psychological outcomes, while those who felt divine closeness experienced greater emotional comfort and motivation. This highlights mindful presence in worship as a key factor linking spiritual practice to psychological adjustment in contexts of displacement and adversity.

It is crucial to emphasize that, from a humanitarian and psychosocial perspective, maintaining religious practice in demanding circumstances—such as a displacement camp—is in itself a significant source of solace and resilience. Theological ideals of focused presence (e.g., *khushū*ʿ) are discussed here as conceptual resources, not as benchmarks against which an individual’s devotion should be measured.

This perspective aligns firmly with a faith-sensitive rather than a faith-based approach to humanitarianism. A faith-sensitive model does not advocate for predefined religious programming or the promotion of any specific practice. Instead, it emphasizes the importance of assessing the spiritual needs and preferences of affected individuals, removing barriers to their chosen practices, and creating safe spaces for observance. Any integration of contemplative elements should occur only when requested by participants and in collaboration with local religious actors, ensuring adherence to humanitarian principles of neutrality, inclusivity, and voluntary participation. This commentary, therefore, does not prescribe a specific Islamic mindfulness protocol but highlights its potential relevance for MHPSS providers to be aware of, should it align with participant-led demand.

## 6 Implications for culturally congruent psychosocial and humanitarian interventions

Rutledge’s (1) findings highlight that the exclusion of faith-sensitive approaches and gender-responsive services in displacement settings exacerbates psychological distress and limits access to effective support for Muslim women affected by conflict. Integrating Islamic mindfulness practices—such as *khushūʿ* in prayer, *dhikr*, *murāqabah*, and *tafakkur*—into psychosocial and humanitarian interventions provides a culturally and spiritually congruent means of addressing stress, fostering resilience, and improving mental-health outcomes. Evidence from programmatic research supports this integration. Evidence from Abdulkerim & Li (35)Anlı (36), and AlBedah et al. (37) indicates that culturally adapted Islamic mindfulness programs outperform standard non-adapted approaches in reducing stress and enhancing well-being that lack religious and cultural adaptation.

Further, interventions that incorporate Qurʾān-based reflection, *dhikr*, and training in mindful attentiveness have been reported as both acceptable and beneficial in conflict-affected Muslim populations (38, 39). These approaches align with international guidelines that call for the inclusion of local faith-sensitive resources in mental-health and psychosocial support (MHPSS) programs to ensure cultural relevance and community acceptance (40). By embedding attentional and coping practices that resonate with the participants’ spiritual frameworks, such interventions can strengthen engagement, reduce barriers to service use, and enhance both psychological and social well-being in humanitarian settings.

Operationally, it is necessary to situate these suggestions within the IASC MHPSS intervention pyramid, which outlines the progression from broad community-based supports to specialized clinical services (41). Within this structure, the integration of tailored contemplative or spiritually oriented practices is more appropriately placed at the focused non-specialized and specialized service levels, where individualized assessment and targeted care can be applied (42). Evidence from evaluations of MHPSS programming indicates that most displaced individuals rely primarily on community-level supports and do not require clinical intervention, while only a smaller subgroup benefits from more intensive forms of care (43). In the upper tiers of the pyramid, providers have the operational space to work in small groups or individually, enabling more precise assessment, attention to gender considerations, and respect for personal preference—elements central to faith-sensitive practice. This structuring maintains the inclusivity and non-prescriptive nature of community-level activities, while allowing for deeper spiritual support when it is requested and clinically and culturally appropriate.

Collectively, the evidence suggests that acknowledging the ethical–spiritual dimensions of coping and integrating culturally congruent Islamic mindfulness into MHPSS and humanitarian programming can improve intervention uptake and outcomes. This approach is particularly important for displaced Muslim women, for whom religious identity and devotional practices are central to meaning-making, resilience, and perceptions of divine care during prolonged adversity.

## 7 Conclusion

This commentary has argued that Rutledge’s (1) findings on faith practice and perceived divine care are clarified by specifying a contemplative dimension within devotion—*khushū*ʿ in prayer, *dhikr*, *murāqabah*, and *tafakkur*—that supports attentional control, emotional regulation, and morally oriented presence. Conceptually, these practices belong to worship rather than clinical technique; analytically, they can be examined for their psychosocial functions without reducing their religious meaning.

Empirically, converging evidence from neurophysiological studies, behavioral measures, and culturally adapted programmes indicates that devotional practices performed with mindful attentiveness are associated with lower stress, improved autonomic balance, and more adaptive appraisals of adversity. For humanitarian settings serving displaced Muslim women, the practical implication is to integrate these familiar practices into faith-sensitive MHPSS in collaboration with local religious actors, ensuring fidelity to religious norms while applying established psychosocial principles.

By situating Islamic mindfulness within the broader framework of faith-sensitive coping, this expansive commentary underscores the value of integrating both ritual and contemplative elements of religious life into future research and intervention design.
